# Sciatic Nerve Palsy following Total Hip Replacement via Direct Anterior Approach after Recommencement of Warfarin for Prophylaxis in Atrial Fibrillation

**DOI:** 10.1155/2014/810481

**Published:** 2014-12-17

**Authors:** Vipin Asopa, Shafic Al-Nammari, Tony Spriggins, Tony Menz, Adrian Bauze

**Affiliations:** Sportsmed.SA, 32 Payneham Road, Stepney, Adelaide, SA 5069, Australia

## Abstract

The occurrence of sciatic nerve palsy following posterior and anterolateral approaches to the hip has been well documented and is about 1-2%. To our knowledge, however, there are no reports of sciatic nerve palsy occurring secondary to the anterior approach to the hip for arthroplasty. We describe a case of sciatic nerve palsy secondary to haematoma formation following total hip replacement through the anterior approach. The recommencement of warfarin for prophylaxis against atrial fibrillation is thought to have been a contributing factor. Full recovery is rare following delayed diagnosis and early recognition of the signs of pain, parasthesia, and gradual loss of dorsiflexion and prompt drainage may reverse the condition. We advise caution with restarting warfarin following total hip arthroplasty.

## 1. Introduction

The incidence of sciatic nerve palsy following total hip replacement through posterior and anterolateral approaches is 0.09–3.7% [1, 2]. Risk factors for the development of sciatic nerve palsy following total hip arthroplasty include developmental dysplasia of the hip, the female sex, posttraumatic arthritis, and revision surgery [1]. The incidence is increased with previous underlying peripheral neuropathy [3].

Sciatic nerve injury can occur at the time of anaesthesia and may be caused by intraneural injection [4], lumbar plexus, or psoas compartment block [5, 6]. Sciatic neuritis can also cause nerve palsy [7]. The intraoperative causes of sciatic nerve injury include significant leg lengthening, improper retractor placement, cement extravasation, cement-related thermal damage, patient positioning, manipulation, and postoperative hematoma [7]. Sciatic nerve palsy can also occur following a closed reduction of a dislocated revision total hip replacement [8].

There are numerous causes for delayed sciatic nerve palsy, including the use of a posterior flange reinforcement ring [9], development of metal debris following primary metal on metal total hip replacement, or wear debris [10, 11]. Two cases of delayed sciatic nerve palsy, occurring 3 weeks and 4 months following primary cement less hip arthroplasty, were thought to have occurred following leg lengthening by 2 cm and 4 cm, respectively [12]. Although we have previously described sciatic nerve palsy due to vascular ischemia arising from intraoperative arterial occlusion [13], to date there are no reports describing sciatic nerve palsy secondary to haematoma after restarting warfarin following arthroplasty through anterior approach to the hip.

## 2. Case

**Figure 1:**
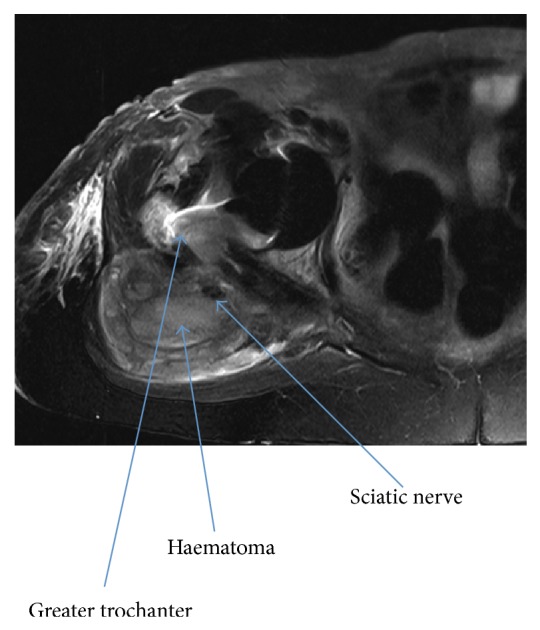
Axial T2 MRI scan showing the haematoma compressing the sciatic nerve.

An 80-year-old female was admitted for a routine total hip replacement. She was previously receiving warfarin for paroxysmal atrial fibrillation and this had been discontinued prior to surgery. A cementless total hip replacement was performed through a direct anterior approach using a Trinity Cluster Shell, a ceramic liner, and a lateralized Metafix femoral stem with ceramic head (Corin, UK). On the evening following uneventful surgery, she was comfortable and had normal neurovascular status. She commenced mobilization and was making good progress the following day. Due to long-standing atrial fibrillation, warfarin was restarted the day after surgery for prophylaxis. Three days after surgery her haemoglobin was 6.8 g/dL and she was transfused 4 units of blood. Overnight she developed severe pain requiring opiate analgesia and she subsequently required another transfusion of 4 units two days later. At this stage, the patient had developed a foot drop and was unable to stand. An MRI scan (Figure 1) demonstrated a large posterior haematoma deep to the iliotibial band and tensor fascia lata. Examination demonstrated numbness below the knee and she was unable to dorsiflex or plantarflex her foot, consistent with a sciatic nerve palsy.

A posterior approach was used to evacuate the posterior haematoma and explore the sciatic nerve. The nerve was in continuity with no evidence of trauma other than compression from overlying haematoma. Gluteus medius was contractile. Haematoma was also evacuated from the anterior wound, and an arthrotomy was performed with lavage of the hip joint. No active bleeding was identified.

Sensation subsequently improved in the lower leg, but she continued to have a foot drop.

## 3. Discussion

The incidence of sciatic nerve palsy secondary to haematoma arising from primary total hip arthroplasty (including posterior approach) is 0.09–3.7% [1]. The use of LMWH has been attributed to an increase in haematoma formation in recent years [2, 14]. Haematoma has also been reported after thrombolysis therapy for acute pulmonary embolism after total hip replacement [14]. In our case, sciatic nerve palsy occurred following haematoma formation after the recommencement of warfarin following anterior approach to the hip. Early recognition of the signs of pain, parasthesia, and gradual loss of dorsiflexion and prompt drainage may reverse the condition [2]. However, full recovery is rare following delayed sciatic nerve palsy [15]. We wish to alert the reader of this potential complication following anterior approach for total hip arthroplasty.
